# Diversity of Peptides Produced by *Nodularia spumigena* from Various Geographical Regions

**DOI:** 10.3390/md11010001

**Published:** 2012-12-21

**Authors:** Hanna Mazur-Marzec, Monika J. Kaczkowska, Agata Blaszczyk, Reyhan Akcaalan, Lisa Spoof, Jussi Meriluoto

**Affiliations:** 1 Department of Marine Biology and Ecology, University of Gdansk, Al. Marszałka Piłsudskiego 46, Gdynia 81-378, Poland; E-Mails: monika.j.kaczkowska@gmail.com (M.J.K.); oceabl@ug.edu.pl (A.B.); 2 Faculty of Fisheries, Istanbul University, Ordu Cad. No. 200, 34470 Laleli, Istanbul, Turkey; E-Mail: akcaalan@istanbul.edu.tr; 3 Department of Biosciences, Abo Akademi University, Tykistökatu 6A, Turku 20520, Finland; E-Mails: lisa.spoof@abo.fi (L.S.); jmeriluo@abo.fi (J.M.)

**Keywords:** *Nodularia spumigena*, cyanobacteria, non-ribosomal peptides, chemotypes, LC-MS/MS

## Abstract

Cyanobacteria produce a great variety of non-ribosomal peptides. Among these compounds, both acute toxins and potential drug candidates have been reported. The profile of the peptides, as a stable and specific feature of an individual strain, can be used to discriminate cyanobacteria at sub-population levels. In our work, liquid chromatography-tandem mass spectrometry was used to elucidate the structures of non-ribosomal peptides produced by *Nodularia spumigena* from the Baltic Sea, the coastal waters of southern Australia and Lake Iznik in Turkey. In addition to known structures, 9 new congeners of spumigins, 4 aeruginosins and 12 anabaenopeptins (nodulapeptins) were identified. The production of aeruginosins by *N. spumigena* was revealed in this work for the first time. The isolates from the Baltic Sea appeared to be the richest source of the peptides; they also showed a higher diversity in peptide profiles. The Australian strains were characterized by similar peptide patterns, but distinct from those represented by the Baltic and Lake Iznik isolates. The results obtained with the application of the peptidomic approach were consistent with the published data on the genetic diversity of the Baltic and Australian populations.

## 1. Introduction

Peptides belong to the most widely studied group of cyanobacterial metabolites. They are characterized by diverse structures and biological activities [1]. Several classes of the peptides, including microcystins, nodularins, aeruginosins, spumigins, anabaenopeptins, microginins and cyanopeptolins, are synthesized by non-ribosomal peptide synthetase (NRPS), or combined NRPS and polyketide synthase (PKS), pathways [2,3,4]. Ribosomal production of peptides has been proposed for cyanobactins [5,6], microviridins [7] and oscillatorins [8]. In some cyanobacteria, both pathways of peptide biosynthesis are active [8].

Non-ribosomal peptide synthetases are multifunctional, large enzyme complexes of a modular structure with catalytic domains responsible for the activation (A), thioestrification (T) and condensation (C) of amino acids or short carboxylic acids into peptidyl compounds [9,10]. These compounds have a linear, cyclic or branched-cyclic structure, and are often composed of non-proteogenic or modified proteogenic amino acids [1]. The most frequent modifications in the structure include heterocyclization, epimerization, methylation, acetylation, halogenation or hydroxylation of the units. Variability in peptide sequences and modifications in the structure of the units result in a high number of peptide congeners [1]. Individual strains of cyanobacteria usually produce more than one class of non-ribosomal peptides (NRPs), with several structural variants within each class. The profile of the peptides is thought to be a specific and stable feature of an individual strain [1]. It can be used to classify cyanobacteria belonging to the same species into metabolically diverse chemotypes. Most frequently, production of the peptides has been reported from *Microcystis*, *Planktothrix *and *Anabaena *genera, e.g., [1,11,12,13,14].

The structural variety of cyanobacterial peptides is reflected in the different biological activities of the compounds. Due to the strong inhibition of eukaryotic protein phosphatases 1 and 2A [15], and the well-recognized hepatotoxic effects, microcystins and nodularins belong to the most widely studied cyanobacterial NRPs. Metabolites, such as cyanopeptolins, anabaenopeptins, aeruginosins, microviridins and micropeptins belong to the inhibitors of serine proteases and other important enzymes [16,17,18,19,20]. Some of the NRPs are the subject of special interest as potential pharmaceuticals showing anticancer, antibacterial, antiviral or anticoagulant activity [21,22,23,24].

The filamentous, nitrogen-fixing cyanobacterium *Nodularia spumigena*, is characterized by the ability to produce nodularin (NOD), a cyclic pentapeptide hepatotoxin of the general structure cyclo [-D-erythro-β-methylAsp(iso-linkage)-L-Arg-Adda-D-Glu(iso-linkage)-2-(methylamino)-2(*Z*) dehydrobutyric acid] where Adda is the C20 β-amino acid, (2*S*,3*S*,8*S*,9*S*)-3-amino-9-methoxy-2,6,8-trimethyl-10-phenyldeca-4(*E*),6(*E*)-dienoic acid (**23** in Table 1) [25,26]. Other NRPs detected in *N. spumigena* belong to the spumigin and nodulapeptin classes [3,4,12,27].

**Table 1:** Peptides identified in *Nodularia spumigena* from the Baltic Sea, Lake Iznik in Turkey and coastal water bodies of Southern Australia. Peptides detected only in bloom sample from the Baltic Sea are marked with asterix (*). AP = anabaenopeptin, NP = nodulapeptin. Black circle—relative peak intensity (*i*) over 1.7 × 10^5^ counts in time-of-flight mass spectra (TOF MS), bigger empty circles—1.0 × 10^4^ < *i* < 1.7 × 10^5^ counts in TOF MS, smaller empty circles—ions of the lowest intensity.

m/z	Oligopeptide structure	Peptide no.	Peptide name	Baltic Sea											Iznik Lake			Australian waters									
				CCNP 1401	CCNP1403	B15a	CCNP1402	KAC66	BY1	CCNP 1423	CCNP 1424		CCNP 1425		Node 2	Nodg 3	Nodh 2	NSBL-05		NSBL-06		NSLA-01	NSOR-02	NSGL-01	NSKR-07	NSBR-01	NSPH-02
				Spumigins																							
655	(Hpla + 42)-Hty-Pro-Arg	1		◦	◦	◦																					
653	(Hpla + 42)-Hty-MePro-Argal	2		◦	◦	◦																					
641	(Hpla + 42)-Hty-Pro-Argol	3		●	●	○																					
639	(Hpla + 42)-Hty-Pro-Argal	4		●	●	●																					
627	Hpla-Hty-MePro-Arg	5	B	◦	◦	◦	◦	◦			◦				◦	◦											
613	Hpla-Hty-MePro-Argol	6	A	○	◦	◦	◦	○	◦						◦	◦	◦										
613	Hpla-Hty-Pro-Arg	7	C	◦	◦	◦		◦														◦		◦			
611	Hpla-Hty-MePro-Argal	8	E	◦	◦	◦	●	◦	◦	◦	◦		◦		◦	◦	◦										
599	Hpla-Hty-Pro-Argol	9	D	◦	◦	◦	◦	◦	◦	◦	◦		◦		◦	◦	◦	○		◦		◦	◦	◦	◦	◦	◦
597	Hpla-Hty-Pro-Argal	10	F	○	○	◦	◦	○	◦	◦	◦		◦		◦	◦	◦	○		◦		◦	◦	○	◦	◦	◦
595	Hpla-Hph-MePro-Argal	11	G				◦		○	◦	◦		◦														
583	Hpla-Tyr-Pro-Argal	12		◦	◦	◦																					
583	Hpla-Hty-MePro-Agm	13													◦	◦	◦										
581	Hpla-Hph-Pro-Argal	14*	H																								
575	(Hpla + 42)-Leu-Pro-Argal	15		◦	◦	◦																					
535	Hpla-Leu-Pro-Argol	16*	I																								
470	Hpla-Hty-MePro-NH_2_	17													◦	◦	◦										
457	Hpla-Hty-Pro-OH	18		◦	◦	◦	◦	◦																			
				Aeruginosins																							
603	-Choi-Arg	19		◦	◦	◦	◦	◦	◦							◦	◦	◦		◦		◦	◦	◦	◦	◦	◦
589	-Choi-Argol	20		◦	◦	◦	○	●	◦						○	◦	◦	◦		◦		◦	◦	◦	○	○	◦
587	-Choi-Argal	21		◦	◦	◦	◦	◦	◦	◦	◦		◦		◦	◦	◦	●		●		●	●	●	●	●	◦
559	-Choi-Agm	22													◦	◦	◦	◦		◦		◦	◦	◦	◦	◦	◦
				Nodularins																							
825	Cyclo[MeAsp-Arg-Adda-Glu-Mdhb]	23	NOD	●	●	●	●	●	●	○	○		○		●	●	●	●					●	●	●	●	●
811	Cyclo[Asp-Arg-Adda-Glu-Mdhb]	24	[dMeAsp^3^]	◦	◦	◦	◦	◦	◦	○	○		○		◦	◦	◦	◦					○	◦	◦	◦	●
			NOD																								
				Anabaenopeptins (Nodulapeptins)																					
934	Phe-CO-[Lys-Val-Hty-MeHty-MetO]	25						◦		○	○	○													
932	Ile-CO-[Lys-MetO-Hph-MeHty-MetO]	26					◦		◦																
930	Ile-CO-[Lys-MetO_2_-Hph-MeHty-AcSer]	27	NP A																						
918	Phe-CO-[Lys-Val-Hph-MeHty-MetO]	28	NP 917					◦		○	○	○													
916	Ile-CO-[Lys-MetO-Hph-MeHty-Met]	29					○		◦					◦	◦	◦									
916	Phe-CO-[Lys-Val-Hty-MeHty-AcSer]	30						◦		○	○	○													
914	Ile-CO-[Lys-MetO-Hph-MeHty-AcSer]	31	NP B				●		●					◦	◦	◦									
902	Phe-CO-[Lys-Val-Hph-MeHty-Met]	32	NP 901					◦		○	○	○													
900	Phe-CO-[Lys-Val-Hph-MeHty-AcSer]	33	NP 899					◦		○	○	○													
900	Ile-CO-[Lys-Met-Hph-MeHty-Met]	34	[Met^6^]				◦		◦																
			NP C																						
898	Ile-CO-[Lys-Met-Hph-MeHty-AcSer]	35	NP C				◦		○					○	○	◦									
898	Ile-CO-[Lys-MetO-Hph-MeHph-AcSer]	36	[MeHph^5^]				●		○					○	○	○									
			NP B																						
884	Ile-CO-[Lys-Met-Hph-MeHph-Met]	37					◦																		
884	Phe-CO-[Lys-Val-Hph-MeHph-AcSer]	38						◦		○	○	○													
882	Ile-CO-[Lys-Met-Hph-MeHph-AcSer]	39					●		◦																
882	Ile-CO-[Lys-Ile-Hph-MeHty-Met]	40					●																		
880	Ile-CO-[Lys-Ile-Hph-MeHty-AcSer]	41					◦		◦					◦	◦	◦									
872	Ile-CO-[Lys-MetO-Hph-MeHty-Ser]	42	[Ser^6^]				◦		◦					◦	◦	◦									
			NP B																						
856	Ile-CO-[Lys-Met-Hph-MeHty-Ser]	43					◦																		
856	Ile-CO-[Lys-MetO-Hph-MeHph-Ser]	44					◦		◦					◦	◦	◦									
842	Phe-CO-[Lys-Ile-Hty-MeAla-Phe]	45		●	●	●																			
828	Phe-CO-[Lys-Val-Hty-MeAla-Phe]	46	AP D	◦	◦	◦																			
808	Ile-CO-[Lys-Ile-Hty-MeAla-Phe]	47															○	○	◦		◦	○	◦	◦	◦
				Unknown peptides																					
829	Unknown	48		○	○	◦		◦	◦					◦	◦	◦									
576	Unknown	49															●	●	●		●	●	●	●	●
562	Unknown	50															◦	◦	○		◦	◦	◦		○

Spumigins are linear tetrapeptides composed of (4-hydroxy-phenyl)lactic acid (Hpla) at the *N*-terminus, homotyrosine (Hty) or homophenylalanine (Hph) in position 2 and proline (Pro) or methylproline (MePro) in position 3 [3,12,27]. The *C*-terminal position is occupied by arginine (Arg) or its mimetics. Due to their structural similarity, spumigins were considered to be a group of aeruginosins [1]. This class of peptides contains Choi^3^ (2-carboxy-6-hydroxyoctahydroindole), instead of Pro^3^. In recent studies, however, a low sequence homology between the gene clusters involved in the biosynthesis of the two classes of peptides (*spu* and *aer*) was shown [3]. Nodulapeptins belong to the class of cyclic hexapeptides called anabaenopeptins. A characteristic feature of all anabaenopeptins is the presence of conserved D-lysine and an ureido bond between Lys and a side chain built of one amino acid [11]. In position 5 of these peptides, *N*-methylated units were reported, while position 4 was most frequently occupied by homo-variants of tyrosine and phenylalanine. The known nodulapeptins have Ser, Met or their derivatives in position 6 [4,12].

In this work, cyanobacterial non-ribosomal peptides, *i.e.*, spumigins, aeruginosins, nodularins and anabaenopeptins, were analyzed in twenty strains of *N. spumigena*. The studied cyanobacteria originated from the Baltic Sea, the coastal waters of southern Australia and the freshwater Lake Iznik in Turkey. The primary aim of the study was to investigate the structural diversity of NRPs in *N. spumigena* and to discover possible novel peptides by tandem mass spectrometry techniques. A further aim was to determine whether the detected oligopeptide profiles might be used as chemotaxonomic features suitable for distinguishing and clustering *N. spumigena* populations. The obtained results were then compared with published data on the genetic diversity of *N. spumigena* strains originating from various geographical regions. The results obtained in this study corroborate the potential of cyanobacteria as a rich source of NRPs, which can be tested for various bioactivities and potentially chosen as lead compounds for drug development.

## 2. Results and Discussion

In this work, the elution profiles of peptides for the 20 strains of *N. spumigena* were determined (Figure S1). The most intense peaks that occurred in the chromatograms of *N. spumigena* isolates are marked in Table 1 with black circle (relative peak intensity (*i*) over 1.7 × 10^5^ counts in TOF MS) or circles with a dot (1.0 × 10^4^ < *i* < 1.7 × 10^5^ counts in TOF MS). In further studies, LC-MS/MS systems were used to characterize the structure of the ions, and also the structure of the less abundant ions. The effectiveness of tandem mass spectrometry in structural elucidation of cyanobacterial peptides has been demonstrated in numerous papers, e.g., [3,4,12,13,14]. The LC-MS/MS method appears to be especially useful in the analysis of small amounts of compounds in complex natural matrices.

The peptides produced by *N. spumigena* were detected as singly protonated ions with *m/z* ranging from 457 to 934. In their fragmentation mass spectra a number of indicative ions, including *b*, *a* and *y* ions, as well as immonium ions, were present. Ions less significant for structure elucidation, but present in the spectra were formed by the loss of water (−18 Da) and carbonyl group (−28 Da). Based on the obtained rich mass fragmentation spectra, as well as the already known sequences of cyanobacterial oligopeptides and their fragment ions, the structures of 47 peptides were elucidated.

The compounds belonged to two classes of linear peptides, spumigins and aeruginosins, and two classes of cyclic peptides, nodularins and anabaenopeptins. Out of the four classes of oligopeptides, nodularins and spumigins were almost exclusively reported from *N. spumigena* (with the exceptions described in [28,29,30]). The compounds belonging to aeruginosins and anabaenopeptins were previously detected in different cyanobacterial taxa, including *Microcystis*, *Planktothrix* and *Anabaena* [1]. In *N. spumigena*, the presence of aeruginosins and anabaenopeptins other than those classified to the nodulapeptin sub-group (*i.e.*, containing Ser or Met in position 6) was revealed in our work for the first time.

### 2.1.Linear Peptides: Spumigins and Aeruginosins

**Figure 1:**
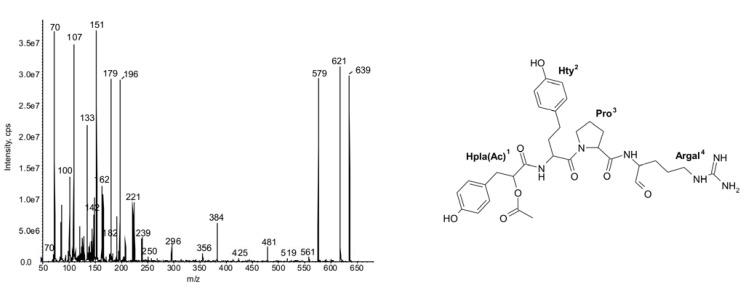
Chemical structure and enhanced ion product mass spectra of spumigin **4** (Hpla + 42)-Hty-Pro-Argal with [M + H] ion at *m/z* 639 (*N. spumigena* B15a). The structure was elucidated mainly based on the mass signals at *m/z*: 621 [M + H − H_2_O], 579 [M + H − H_2_O − CH_2_N_2_] or/and [M + H − CH_3_COOH], 481 [(Hpla + 42)-Hty-Pro + H], 384 [(Hpla + 42)-Hty + H], 356 [(Hpla + 42)-Hty + H − CO], 250 [AcOCHCO-Hty + H − CO], 239 [Pro-Argal + H − NH_2_], 221 [Pro-Argal + H − H_2_O − NH_2_], 142 [Argal + H − NH_3_], 107 [CH_2_PhOH], 100 [C_4_H_10_N_3_] Argal fragment, 70 Pro-immonium ion.

In *N. spumigena*, a total of 18 spumigins were detected, including nine new congeners of the compounds (Table 1, **1**–**4**, **12**, **13**, **15**, **17**, **18**). The elucidation of spumigin structures was based on the fragmentation pattern in mass spectra described by [3,12,27]. All spumigins reported so far have Hpla as the *N*-terminal unit [3,27]. Also, in the majority of spumigins detected in this work, Hpla^1^ was present. However, in compounds **1**–**4**, instead of an ion at *m/z* 208 [HOCHCO-Hty + H − CO], a fragment ion at *m/z *250 was recorded (Figure 1).

The shift in mass (42 Da) was also observed in other fragment ions containing the *N*-terminal unit. This mass difference may indicate the presence of an acetyl group in the structure of Hpla. Such a modification was found in spumigins produced by three strains from the Gulf of Gdańsk (CCNP1401, B15a and CCNP1403). In these isolates, the modified spumigins belonged to the most abundant peptides (Table 1).

The second position in the spumigins structure was the least conserved. It was mainly occupied by Hty^2^ (13/18), but in individual peptides, homophenylalanine Hph^2^ (**11** and **14**), Leu^2^ (**15 **and **16**) or Tyr^2^ (**12**) were also incorporated. Spumigin H (**14**) with Leu^2^ and spumigin I (**16**) with Hph^2^ were found only in *N. spumigena* bloom samples from the Baltic Sea (Table 1). In position 3 of the spumigins, the presence of Pro^3^ (11/18) or MePro^3^ (7/18) was deduced based on the enhanced ion product (EIP) spectra with immonium ions (at *m/z* 70 or 84) and a series of other ions containing Pro or MePro. This result confirmed a preferential activation of Pro (or MePro) by the catalytic domain of the enzyme responsible for the incorporation of the third residue during the biosynthesis of spumigins in *N. spumigena* CCY9414 [3].

The *C*-terminal position of the spumigins was occupied by Arg (**1**, **5**, **7**), its decarboxylated form called agmatine (Agm: **13**), the alcohol form (Argol: **3**, **6**, **9**, **16**) or the aldehyde form (Argal: **2**, **4**, **8**, **10**–**12**, **14**, **15**). Spumigins with Argal were the most common and belonged to the most abundant congeners (Table 1). In the chromatograms, they occurred as broad, tailing and/or multiple peaks, indicating the presence of different isomers. The high prevalence of Argal in the *C*-terminal position confirmed the previous results on the structure of the multifunctional enzyme complex involved in the biosynthesis of spumigins in *N. spumigena* CCY9414. Fewer *et al.* [3] revealed that the module of the complex responsible for the incorporation of the *C*-terminal unit, apart from the Arg-activating A domain, contains a reductase domain, which leads to the production of spumigins as peptide aldehydes.

In the EIP spectrum of compound **18** with a molecular ion at *m/z* 457, the presence of ions at *m/z* 342 [Hpla-Hty + H], 439 [Hpla-Hty-Pro + H] was indicative of the spumigin structure (see Supplementary Information). This spumigin congener was found in five of the Baltic isolates. As the mass difference between the [M + H]^+^ ion of compound **18** and [Hpla-Hty-Pro + H] was calculated to be 18 Da, we proposed that the structure of compound **18** is Hpla-Hty-Pro-OH. The other tripeptide with a molecular ion at *m/z* 470 (**17**) and fragment ions at *m/z* 342 [Hpla-Hty + H], 453 [Hpla-Hty-MePro + H] and 84 (MePro-immonium ion), was identified as Hpla-Hty-MePro-NH_2_. In our work, compound **17** was found in the cyanobacteria isolated from Lake Iznik and in a bloom sample from the Baltic Sea (Table 1). The tripeptides of general structure Hpla-Leu-Choi-NH_2_, aeruginosin 298B [16] and aeruginosin EI461 [31] were identified in *Microcystis aeruginosa* and suggested as biosynthetic intermediates of the respective tetrapeptides.

Aeruginosins are composed of Choi^3^ (2-carboxy-6-hydroxyoctahydroindole) and Arg^4^ or its mimetics [1,2,16,32,33]. In the second position of aeruginosins characterized in previous studies, variable amino acids were found: Tyr, Hty, Phe, Leu or Ile [1]. In most of the published structures of the peptides, the *N*-terminal position was occupied by Hpla^1^ [16]. Pla (phenyl lactic acid) in position 1, though rarely reported, was found in oscillarin, one of the most potent thrombine inhibitors among aeruginosin analogues [34]. Additionally, chlorinated and/or sulphated aeruginosins (at both Hpla and Choi) as well as compounds with a pentose sugar were detected [13,14,16,23,35].

In our studies, production of aeruginosins by *N. spumigena* isolates, regardless of their origin, was revealed for the first time. The partial reconstruction of the amino acid sequence in the four detected variants (Table 1, **19**–**22**) was conducted with the support of the data published by [13,14,35,36], among others. Like all spumigins identified in *N. spumigena* cells, the peptides did not contain any chloride or sulphate groups. 

**Figure 2:**
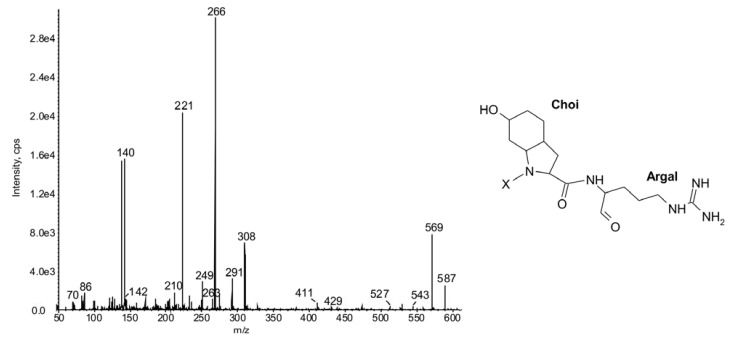
Chemical structure and enhanced ion product mass spectra of partially identified aeruginosin **21** with [M + H] ion at *m/z* 587 (*N. spumigena* KAC66). The mass signals were assigned to the following fragments: 569 [M + H − H_2_O], 527 [M + H − H_2_O − CH_2_N_2_], 429 [M + H − Argal], 411 [M + H − Argal − H_2_O], 308 [Choi-Argal + H − H_2_O − CH_3_N_2_], 291 [Choi-Argal + H − H_2_O − NH_3_], 266 [Choi-Argal + H − H_2_O − CH_2_N_2_], 140 Choi-immonium ion, 142 [Argal + H − NH_3_]. X—unknown part of the molecule.

As in spumigins, the *C*-terminal position of aeruginosins detected in this study was occupied by Arg (**19**), Argol (**20**), Argal (**21**) or Agm (**22**) (Table 1). Under the MS/MS conditions, the EIP spectra of aeruginosins were significantly affected by the unit. When Argal was incorporated in the *C*-terminal position, the ions at *m/z* 221 and 266 belonged to the most intense ions in the sequence (Figure 2).

In the case of aeruginosins with Arg, Argol and Agm, the other two ions corresponding to fragments [Choi-Arg/Argol/Agm + H] and [Choi-Arg/Argol/Agm + H − NH_2_] were more abundant (see Supplementary Information). Compound **22** with Agm was found in all Australian isolates and the strains from Lake Iznik in Turkey. Similarly to Agm-containing spumigins, compound **22** was not present in either the Baltic bloom samples or the Baltic *N. spumigena* isolates. This finding might indicate the differences in the non-ribosomal peptide synthetases among *N. spumigena* populations from different regions.

### 2.2. Cyclic Peptides: Nodularins and Anabaenopeptins

**Figure 3:**
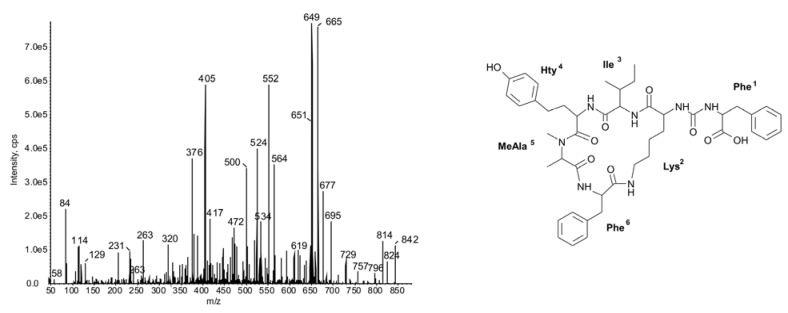
Chemical structure and enhanced ion product mass spectra of anabaenopeptin **45** Phe-CO-[Lys-Ile-Hty-MeAla-Phe] with [M + H] ion at *m/z* 842 (*N. spumigena* CCNP1401). The mass signals were assigned to the following fragments: 824 [M + H − H_2_O], 814 [M + H − CO], 796 [M + H − H_2_O − CO], 757 [M + H − MeAla], 729 [M + H − Ile], 677 [M − Phe − 2H], 665 [M + H − Hty], 651 [M + H − (CO-Phe)], 649 cyclo[Lys-Ile-Hty-MeAla-Phe − H], 552 [M + H − (Hty-Ile)], 534 [M + H − (Hty-Ile) − H_2_O], 405 [MeAla-Phe-(Lys-CO) + H], 376 [Ile-Hty-MeAla + H], 320 [(Lys-CO-Phe) + H], 263 [MeAla-Hty + H], 231 [MeAla-Phe − H], 114 [MeAla-CO + H], 84 Lys-immonium ion, 58 MeAla-immonium ion.

In *N. spumigena*, two classes of cyclic NRPs were identified: the toxic pentapeptides called nodularins (**23** and **24**) and the hexapeptides classified as anabaenopeptins (**25**–**47**). The hepatotoxic nodularins belong to the most widely studied cyanobacterial metabolites [37]. With the exception of *N. spumigena* NSBL-05 and NSBL-06, the peak of NOD was one of the most intense in the obtained chromatograms. The lack of NOD and its demethylated form [D-Asp^3^]NOD in the two Australian strains (NSBL-05 and NSBL-06) is in agreement with the results of Bolch *et al.* [38] and Moffitt *et al.* [39], who found these strains to be non-toxic. Out of the 23 anabaenopeptins, twelve variants have been reported in this work for the first time (**25**, **26**, **30**, **37**–**41**, **43**–**45**, **47**). Their structures were elucidated using the published data on anabaenopeptins mass fragmentation spectra [4,12,40,41,42,43]. As in all other compounds classified as anabaenopeptins, homo amino acids (Hph and Hty) were present in position 4, and *N*-methylated amino acids (methylhomotyrosine MeHty, methylhomophenylalanine MeHph and methylalanine MeAla) in position 5 (Figure 3).

**Figure 4:**
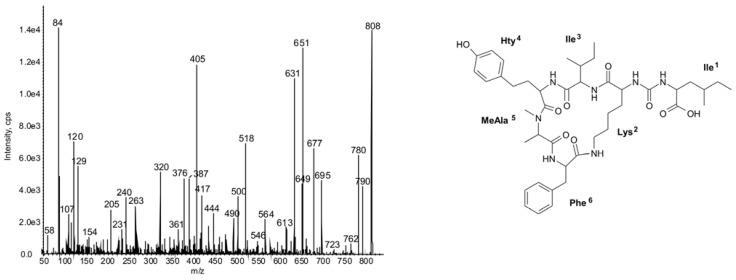
Chemical structure and enhanced ion product mass spectra of anabaenopeptin **47** Ile-CO-[Lys-Ile-Hty-MeAla-Phe] with [M + H] ion at *m/z* 808 (*N. spumigena* NSOR-02). The mass signals were assigned to the following fragments: 790 [M + H − H_2_O], 780 [M + H − CO], 762 [M + H − H_2_O − CO], 695 [M + H − Ile], 677 [M + H − H_2_O − Ile], 651 [M + H − (CO-Ile)], 649 cyclo[Lys-Ile-Hty-MeAsp-Phe − H], 631 [M + H − Hty], 546 [Phe-(Lys-CO-Ile)-Ile + H], 518 [M + H − (Hty-Ile)], 500 [M + H − H_2_O − (Hty-Ile)], 417 [Phe-(Lys-CO)-Ile + H], 405 [MeAla-Phe-(Lys-CO) + H], 376 [Ile-Hty-MeAla + H], 361 [MeAla-Lys-Phe + H], 263 [Hty-MeAla + H], 231 [MeAla-Phe − H], 120 Phe-immonium Ion, 107 [CH_2_PhOH], 84 Lys-immonium ion, 58 MeAla-immonium ion.

In position 3, Met or its oxidized forms (methionine sulfoxide MetO and methionine sulfone MetO_2_), were the most common residues (12/23). In other compounds the position was occupied by Val (7/23) or Ile/Leu (4/23). Position 6 was the least conserved and, as in all reported nodulapeptins, it contained Ser or its acetylated form AcSer (*O*-acetyl Ser) (12/23), or Met or its oxidized form MetO (8/23). Additionally, we revealed the production of three anabaenopeptins with Phe in position 6 (Figure 3 and Figure 4). In the side chain unit, either Phe^1^ (Figure 3) or Ile/Leu^1^ (Figure 4) was incorporated. In *N. spumigena* strain KAC66, production of nodulapeptins 899 (**33**), 901 (**32**) and 917 (**28**) with Phe in position 1 was reported by Schumacher *et al.* [43]. These peptides and three other nodulapeptins (**25**, **30** and **38**), all containing Phe^1^ in their backbone chain, were also detected in KAC66 in our study.

Based on the mass spectrometrically elucidated structures studied, it can be concluded that individual strains of *N. spumigena* produced anabaenopeptins with only one kind of amino acid in the exocyclic position, *i.e.*, either Ile/Leu or Phe. This result is in agreement with the data published by Rouhiainen *et al.* [4] who found only one starter module in the anabaenopeptin synthetase gene cluster (*apt*) of *N. spumigena* CCY9414. The A domain of the module probably activates only Ile or only Phe. In contrast, the *apt* gene clusters in *Anabaena *encode two alternative starter modules organized in separate bimodular proteins [4]. Such organization of anabaenopeptin gene clusters or the substrate promiscuity of the A domain [44], lead to the co-production of anabaenopeptins with more than one unit in the exocyclic position 1.

According to the co linearity rule, the order and the number of the modules in the NRPS correspond to the number and sequence of the units in the biosynthesized peptide [9]. Therefore, based on the organization of the NRPS, and the substrate specificity of adenylation domains, the structure and diversity of the peptide products can be predicted. The reverse process is also possible. The analyses of the whole variety of non-ribosomal peptide congeners produced by strains belonging to the same species can be a valuable source of information about the structure of the NRPS enzyme complex. The chemical structures of spumigins and anabaenopeptins elucidated in 20 strains of *N. spumigena* from the Baltic Sea, coastal waters of southern Australia and freshwater Lake Iznik corresponded well with the published data on the organization of their respective gene clusters (*spu* and *apt*) and the non-ribosomal peptide synthetases enzyme complexes in *N. spumigena* CCY9414 [3,4].

### 2.3. Diversity in Peptidomic Profiles of *N. spumigena* Strains

Despite the differences in the phenotypic features of individual *N. spumigena* strains, there is a high percentage of similarity in their 16S rRNA gene sequences [39,45]. The genetic studies by Hayes and Barker [46] and Moffitt *et al.* [39] showed a lack of delineation between *N. spumigena* from geographically distant regions. A contradictory hypothesis was presented by Bolch *et al.* [38] based on the analysis of the phycocyanin intergenic spacer region (*cpcBA*-IGS). This highly variable region is thought to be a useful molecular marker for discrimination of cyanobacterial strains. For all Australian isolates, including the non-toxic NSBL, the *cpcBA*-IGS sequences were identical, but different from the Baltic populations [38]. The genetic difference between the Baltic and Australian strains was also documented by Lehtimäki *et al.* [45] using the RFLP (restriction fragment polymorphism) of 16S rRNA genes and other molecular techniques.

**Figure 5:**
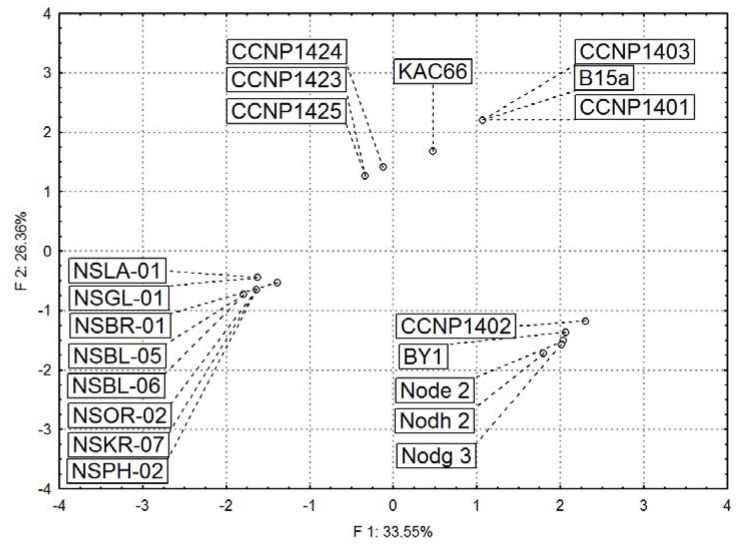
Principal component analysis (PCA) ordination plots (F1 × F2) of *Nodularia spumigena* strains based on their peptide content as analyzed by LC-MS/MS.

The grouping of *N. spumigena* strains based on the analyses of peptide profiles appears to be consistent with the genetic diversity of the cyanobacterium as determined by analysis of the nucleotide sequences of the *cpc*BA-IGS fragments. The principal component analyses of 50 peptides in the 20 *N. spumigena *strains placed all the Australian isolates in one group (Figure 5). This group included both nodularin-producing and “non-toxic” (NSBL) *N. spumigena* strains, and was distantly related to the strains from other geographical regions.

In addition, both the chemical analyses performed in this study and the published data on the PC-IGS sequences [38,45,47] indicated the existence of several distinct types of the Baltic *N. spumigena*. On the factorial plane (Figure 5), the Baltic strains were distributed widely and represented different chemotypes. Two of them, BY1 and CCNP1402, were classified to one chemotype together with *N. spumigena* from the freshwater Lake Iznik in Turkey (Node2, Nodg3 and Nodh2) (Figure 5). The fact that strains from such diverse environments and distinct geographical regions showed similar peptide patterns indicates that environmental conditions, or at least salinity, have no effect on the production of the metabolites. 

Significant variations in the number of cyanobacterial chemotypes among different reservoirs have been reported for *Planktothrix agardhii* [13,48,49,50] and *Microcystis aeruginosa* [14,51,52]. So far, the mechanisms regulating the number and composition of cyanobacterial chemotypes in aquatic ecosystems have not been recognized. It was postulated that the diversification of oligopeptide profiles is driven by horizontal gene transfer, recombination events (especially within the adenylation domains) or gene deletion [4,32,53,54,55]. The significance of natural selection affected by environmental pressure has also been taken into account.

Despite extensive studies on cyanobacterial NRPs, the ecological function of the compounds is still ambiguous. The peptides were suggested to play some role in competition with eukaryotic algae [56], or as defense agents against grazers [18,19]. Alternatively, they might belong to signaling molecules in quorum sensing mechanisms [57]. The potential role of the compounds in cyanobacteria bloom termination through viral lysis [58] or in the interaction with chytrid fungi infecting cyanobacteria [59] was also considered.

*N. spumigena *appeared to be an effective producer of NRPs. Several classes of the peptides, including those analyzed in our study, inhibit the activity of important metabolic enzymes and are considered to be potential therapeutic agents. Fewer *et al.* [3] revealed a strong (nM) trypsin inhibition of spumigin E (**8**). Aeruginosins were revealed to exhibit high *in vitro* inhibitory activity against blood coagulation factors (thrombin, trypsin, Factor VIIa and Factor IXa) [16,21,22]. The activity of anabaenopeptins towards protein phosphatases [60] and proteolytic enzymes such as trypsin, chymotrypsin, elastase and carboxypeptidase [61,62,63] was also demonstrated.

## 3. Experimental Section

### 3.1. Source of Cyanobacteria and Culture Conditions

Twenty strains of *N. spumigena* were analyzed. Seven of them were isolated from the Gulf of Gdańsk, southern Baltic, during the summer bloom of cyanobacteria: CCNP1401, CCNP1403 and B15a in 1997, CCNP1402 in 2005 and CCNP1423, CCNP1424 and CCNP1425 in 2011. Two other Baltic strains were obtained from culture collections: KAC66 isolated from Landsort Deep in 1996 (Kalmar Algae Collection, University of Kalmar, Kalmar, Sweden) and BY1 isolated from Arkona Sea in 1986 (Department of Applied Chemistry and Microbiology, Helsinki University, Helsinki, Finland). Three *N. spumigena* strains, Node2, Nodg3 and Nodh2, were isolated in 2005 from the freshwater Lake Iznik located in the southeast of the Marmara region in Turkey. Eight Australian strains were provided by the CSIRO Marine Laboratories (Hobart, Tasmania, Australia): NSBL-05 and NSBL-06 (Lake Bullenmerri, Victoria, Australia, 1993), NSGL-01 (Gippsland Lake, Victoria, Australia,1993), NSLA-01 (Lake Alexandrina, South Australia, Australia, 1990), NSOR-01 (Orielton Lagoon, Tasmania, Australia, 1993), NSKR-07 (Kalgan River Estuary, Western Australia, Australia, 1995), NSBR-02 (Blackwood River, Western Australia, Australia, 1994), and NSPH-02 (Peel-Harvey Inlet, Western Australia, Australia, 1992).

Cyanobacteria were grown in a Z8 medium (without nitrogen) prepared using MilliQ water (PF system, Millipore, Molsheim, France) with NaCl added to produce a final salinity of 7 psu. Cultures were incubated for approximately four weeks in growth chambers at 22 °C and irradiance of 20 μmol photons m^−2^ s^−1^ with a 16:8 light:dark cycle. Cyanobacteria were harvested when the stationary phase of growth was reached and the optical density of cell suspension was about 0.230 (at 750 nm). Additionally, field samples were collected during blooms of *N. spumigena* in the Gulf of Gdańsk on 30 June 2011 and 3 July 2012. The analyzed *N. spumigena* strains possessed discoid vegetative cells (mean width 5.22–7.05 μm; mean length 2.87–4.68 μm), with gas vesicles. Heterocytes were more spherical (mean width 6.38–8.12 μm; mean length 4.40–7.67 μm), and occurred predominantly in intercalary positions in straight or curved filaments.

### 3.2. Extraction and LC-MS/MS Analyses

*N. spumigena* cultures (30 mL) or bloom samples (100 mL) were filtered onto GF/C glass-fiber filters (Whatman). The material was extracted with 2.0 mL of 5% acetic acid in MilliQ water by a 1 min probe sonication with an ultrasonic disrupter (HD 2070 Sonopuls, Bandeline, Berlin, Germany) followed by a 15 min bath sonication (Sonorex, Bandeline, Berlin, Germany). The samples were then centrifuged at 10,000 g for 15 min and the obtained supernatants were subjected to LC-MS/MS analysis.

*N. spumigena* cell extracts were first screened for peptides using a nano-LC (nanoAquity, Waters, Milford, MA, USA) coupled to a QStar Elite hybrid quadrupole-time-of-flight tandem mass spectrometry system (Q-TOF MS/MS, Applied Biosystems MDS Sciex, Concord, ON, Canada). The analyses were conducted using a NanoSpray II source and Heated Interface (150 °C) on the QSTAR Elite LC-MS/MS. Chromatography was performed with 5% acetonitrile in MilliQ water plus 0.1% formic acid (solvent A) and 0.1% formic acid in acetonitrile (solvent B). Samples (0.5 μL) were injected on a nanoAquity UPLC Symmetry C18 trapping column (180 μm × 20 mm, 5 μm, Waters, Milford, MA, USA) and washed with solvent A at a flow rate of 15 μL min^−1^ for 2 min. Peptides were separated on a nanoAquity UPLC BEH130 C18 column (75 μm × 100 mm, 1.7 μm, Waters, Milford, MA, USA). The gradient started at 5% B and went to 30% B within 30 min at a flow rate of 0.4 μL min^−1^. The content of phase B was then increased to 99% within the next 15 min and kept at that level for 10 min before returning to the starting conditions. Compounds eluting from the column were ionized using a PicoTip emitter (10 μm tip I.D.; New Objective, Woburn, MA, USA). MS spectra were acquired over the range 350–2000 Da. The instrument was operated in the positive mode. Ionspray voltage was 2.4 kV, with the nebulizing gas nitrogen pressure and curtain gas nitrogen pressures set at 25 p.s.i and 20 p.s.i., respectively (1 p.s.i. = 6894.76 Pa). When the ion product (IP) mode was used, the collision energy (CE) was 55 eV and mass range 50–1000 Da. The system was calibrated with a solution containing synthetic peptide ALILTLVS and CsJ (5 × 10^−7^ M) in 50% aqueous methanol plus 0.1% formic acid. Data acquisition and processing were accomplished with the Analyst^®^ QS 2.0 software.

Structural analyses of selected cyanobacterial peptides were also performed using Agilent 1200 (Agilent Technologies, Waldboronn, Germany) coupled online to a hybrid triple quadrupole/linear ion trap mass spectrometer (QTRAP5500, Applied Biosystems, Sciex; Concorde, ON, Canada). As a mobile phase a mixture of A (5% acetonitrile in MilliQ water plus 0.1% formic acid) and B (0.1% formic acid in acetonitrile) was used. Separation was performed on a Zorbax Eclipse XDB-C18 column (4.6 × 150 mm; 5 μm) (Agilent Technologies, Santa Clara, CA, USA). Phase B was linearly increased from 15% to 75% in 5 min and then to 90% in the next 5 min. This composition of the mobile phase was held for 5 min and brought back to 15% B in 1 min. The column oven temperature was 35 °C, the flow rate was 0.6 mL·min^−1^ and the injection volume was 5 μL. Turbo ion spray (550 °C) voltage was 5.5 kV, with the nebulizer gas pressure and curtain gas pressures set at 60 p.s.i. and 20 p.s.i., respectively.

To characterize the structure of cyanobacterial peptides with the QTRAP LC-MS/MS system, the experiments were run using the information dependent acquisition method (IDA) and in enhanced ion product mode (EIP). In EIP mode, the ions fragmented in the collision cell (Q2) were captured in the ion trap and then scanned. In the IDA method, Q3 survey scans were used to automatically trigger an EIP scan if the signal was above a threshold of 100,000 cps. EPI spectra were acquired from 50 to 1000 Da with a scan speed of 2000 Da s^−1^ and a collision energy (CE) of 45 V with collision energy spread (CES) of 20 V. The linear ion trap fill time was 50 msec. The dynamic exclusion was activated to minimize the risk of missing the co-eluting compounds. Data acquisition and processing were accomplished using Analyst QS^®^ 1.5.1 software.

LC-MS/MS analyses were performed for each strain at least five times using extracts obtained from different culture experiments conducted during two years of the study.

### 3.3. Statistical Analyses

The principal component analysis (PCA) ordination was performed to study the differences in peptide pattern among 20 *N. spumigena* strains from various geographical regions. PCA was generated using the multivariate data analysis software STATISTICA v.9 (StatSoft, Tulsa, OK, USA). In the analyses, all detected peptides (*n* = 50) and the relative intensities of respective mass signals were included.

## 4. Conclusions

With the exception of the Australian strains isolated from Lake Bullenmerri, *Nodularia spumigena* produced compounds belonging to four classes of non-ribosomal peptides: spumigins, aeruginosins, nodularins and anabaenopeptins. Within each class of the peptides, various isoforms were identified. The structures of spumigins and anabaenopeptins elucidated in 20 analyzed strains corresponded well to the existing knowledge of the organization of respective non-ribosomal peptide synthetase gene clusters in *N. spumigena* CCY9414 [3,4]. The studies revealed a high similarity of peptide profiles in the Australian strains and significant diversity of the Baltic *N. spumigena* chemotypes. The obtained results were consistent with the published data on the genetic diversity of the Baltic and Australian *N. spumigena* based on the sequence analysis of *cpc*BA-IGS [37,44]. So far, the factors regulating the composition of cyanobacterial chemotypes in aquatic ecosystems have not been recognized.

The strain-specific differences in the NRPs production should be seriously considered when screening for bioactive compounds for potential pharmaceutical applications.

## Supplementary Files

Supplementary File 1 Supplementary Information (PDF, 471 KB)
