# Supplementary material for: Diversity of Peptides Produced by Nodularia spumigena from Various Geographical Regions
Source: Mar Drugs. 2012 Dec 21;11(1):1–19. doi: 10.3390/md11010001 (PMC3564153; doi:10.3390/md11010001)
Supplement: Supplementary File 1 — Supplementary Information (PDF, 471 KB) [file marinedrugs-11-00001-s001.pdf]

Supplementary Information

Table of Contents

Figure S1. Total ion current chromatograms of *N. spumigena* strains: NSBL-06 (**A**), NSBR-02 (**B**), CCNP1401 (**C**) and BY1 (**D**). 3

Figure S2. Enhanced ion product mass spectra of spumigin **12** Hpla-Tyr-Pro-Argal with [M + H] ion at *m/z* 583 (*N. spumigena* 1403). The structure was elucidated mainly based on the mass signals at *m/z*: 565 [M + H − H_2O], 523 [M + H − H_2O − CH_2N_2], 425 [Hpla-Tyr-Pro + H], 300 [Hpla-Tyr + H − CO], 239 [Pro-Argal + H − NH_2], 221 [Pro-Argal + H − H_2O − NH_2], 142 [Argal + H − NH_3], 136 Tyr-immonium ion, 100 [$C_4H_{10}N_3$] Argal fragment, 70 Pro-immonium ion. 4

Figure S3. Enhanced ion product mass spectra of spumigin **13** Hpla-Hty-MePro-Agm with [M + H] ion at *m/z* 583 (*N. spumigena* Nodh2). The structure was elucidated mainly based on the mass signals at *m/z*: 565 [M + H − H_2O], 541 [M + H − CH_2N_2], 523 [M + H − H_2O − CH_2N_2], 453 [Hpla-Hty-MePro + H], 342 [Hpla-Hty + H], 314 [Hpla-Hyr + H − CO], 225 [MePro-Agm + H − NH_2], 208 [HOCHCO-Hty + H − CO], 114 [$C_5H_{12}N_3$] (Agm), 107 [CH_2PhOH], 84 MePro-immonium ion. 5

Figure S4. Enhanced ion product mass spectra of spumigin **15** (Hpla + 42)-Leu-Pro-Argal with [M + H] ion at *m/z* 575 (*N. spumigena* B15a). The structure was elucidated mainly based on the mass signals at *m/z*: 557 [M + H − H_2O], 515 [M + H − H_2O − CH_2N_2] or/and [M + H − CH_3COOH], 417 [(Hpla + 42)-Leu-Pro + H], 320 [(Hpla + 42)-Leu + H], 292 [(Hpla + 42)-Leu + H − CO], 239 [Pro-Argal + H − NH_2], 221 [Pro-Argal + H − H_2O − NH_2] 142 [Argal + H − NH_3], 100 [$C_4H_{10}N_3$] Argal fragment, 86 Leu-immonium ion, 70 Pro-immonium ion. 6

Figure S5. Enhanced ion product mass spectra of spumigin **18** Hpla-Hty-Pro-OH with [M + H] ion at *m/z* 457 (*N. spumigena* CCNP1403). The structure was elucidated mainly based on the mass signals at *m/z*: 439 [Hpla-Hty-Pro + H], 342 [Hpla-Hty + H], 314 [Hpla-Hyr + H − CO], 208 [HOCHCO-Hty + H − CO], 107 [CH_2PhOH], 70 Pro-immonium ion. 7

Figure S6. Enhanced ion product mass spectra of partially identified aeruginosin **19** with [M + H] ion at *m/z* 603 (*N. spumigena* KAC66). The mass signals were assigned to the following fragments: 585 [M + H − H_2O], 429 [M + H − Arg], 342 [Choi-Arg + H], 325 [Choi-Arg + H − NH_2], 307 [Choi-Arg + H − H_2O − NH_3], 300 [Choi-Arg + H − CH_2N_2]), 282 [Choi-Arg + H − H_2O − CH_2N_2], 175 [Arg + H], 140 Choi-immonium ion, 122 Choi-immonium − H_2O. 8

Figure S7. Enhanced ion product mass spectra of partially identified aeruginosin **22** with [M + H] ion at *m/z* 559 (*N. spumigena* Node2). The mass signals were assigned to the following fragments: 541 [M + H − H_2O], 298 [Choi-Agm + H], 281 [Choi-Agm + H − NH_2], 256 [Choi-Arg + H − CH_2N_2], 238 [Choi-Arg + H − H_2O − CH_2N_2], 210 [Choi-Arg + H − H_2O − CH_2N_2 − CO], 140 Choi-immonium ion, 122 Choi-immonium − H_2O, 114 [$C_5H_{12}N_3$] (Agm). 9

Figure S8. Enhanced ion product mass spectra of anabaenopeptin **25** Phe-CO-[Lys-Val-Hty-MeHty-MetO] with [M + H] ion at *m/z* 934 (*N. spumigena* bloom sample, 3 July 2012). The mass signals were assigned to the following fragments: 916 [M + H − H_2O], 906 [M + H − CO], 888 [M + H − H_2O − CO], 870 [M + H − CH_3SOH (from MetO)], 787 [M + H − MetO], 769 [M + H − Phe − 2H], 757 [M + H − Hty], 705 [M + H − Phe − 2H − CH_3SOH (from MetO)], 677 [M + H − (CO-Phe) − 2H − CH_3SOH (from MetO)], 594 [Val-(Lys-CO)-MetO-MeHty + H], 511 [MeHty-MeO-(Lys-CO) + H], 447 [MeHty-MeO-(Lys-CO) + H − CH_3SOH (from MetO)], 403 [Lys-Val-Hty-H], 369 [MeHty-Hty + H], 339 [MetO-MeHty + H], 164 MeHty, 107 [CH_2PhOH], 84 Lys-immonium ion. 10

Figure S9. Enhanced ion product mass spectra of anabaenopeptin **32** Ile-CO-[Lys-MetO-Hph-MeHty-MetO] with [M + H] ion at *m/z* 932 (*N. spumigena* CCNP1402). The mass signals were assigned to the following fragments: 914 [M + H − H_2O], 904 [M + H − CO], 886 [M + H − H_2O − CO], 868 [M + H − CH_3SOH (from MetO)], 801 [M + H − Ile − 2H], 737 [M + H − Ile − 2H − CH_3SOH (from MetO)], 511 [MeHty-MeO-(Lys-CO) + H], 447 [MeHty-MeO-(Lys-CO) + H − CH_3SOH (from MetO)], 436 [MeHy-Hph-MetO + H − CH_3SOH (from MetO)], 339 [MeHty-MetO + H], 164 MeHty, 107 [CH_2PhOH], 84 Lys-immonium ion. 11

Figure S10. Enhanced ion product mass spectra of anabaenopeptin **39** Ile-CO-[Lys-Met-Hph-MeHph-AcSer] with [M + H] ion at *m/z* 882 (*N. spumigena* BY1). The mass signals were assigned to the following fragments: 864 [M + H − H_2O], 854 [M + H − CO], 822 [M + H − CH_3COOH (from AcSer)], 751 [M + H − Ile − 2H], 725 [M + H − (CO-Ile)], 723 [M + H − (CO-Ile) − 2H], 530 [Met-(Lys-CO-Ile)-Met + H − H_2O], 486 [Met-(Lys-CO-Ile)-(AcSer) + H − CH_3COOH (from AcSer)], 477 [MeHph-AcSer-(Lys-CO) + H], 468 [MeHph-Hph-Met + H], 337 [Hph-MeHph + H], 84 Lys-immonium ion. 12

Figure S11. Enhanced ion product mass spectra of anabaenopeptin **41** Ile-CO-[Lys-Ile-Hph-MeHty-AcSer] with [M + H] ion at *m/z* 880 (*N. spumigena* Node2). The mass signals were assigned to the following fragments: 862 [M + H − H_2O], 852 [M + H − CO], 834 [M + H − H_2O − CO], 820 [M + H − CH_3COOH (from AcSer)], 767 [M + H − Ile], 749 [M + H − Ile − 2H], 723 [M + H − (CO + Ile)], 719 [M + H − Hph], 606 [MeHty-AcSer-(Lys-CO-Ile) + H], 493 [MeHty-AcSer-(Lys-CO) + H], 164 MeHty, 107 [CH_2PhOH], 84 Lys-immonium ion. 13

Figure S1. Total ion current chromatograms of *N. spumigena* strains: NSBL-06 (**A**), NSBR-02 (**B**), CCNP1401 (**C**) and BY1 (**D**).

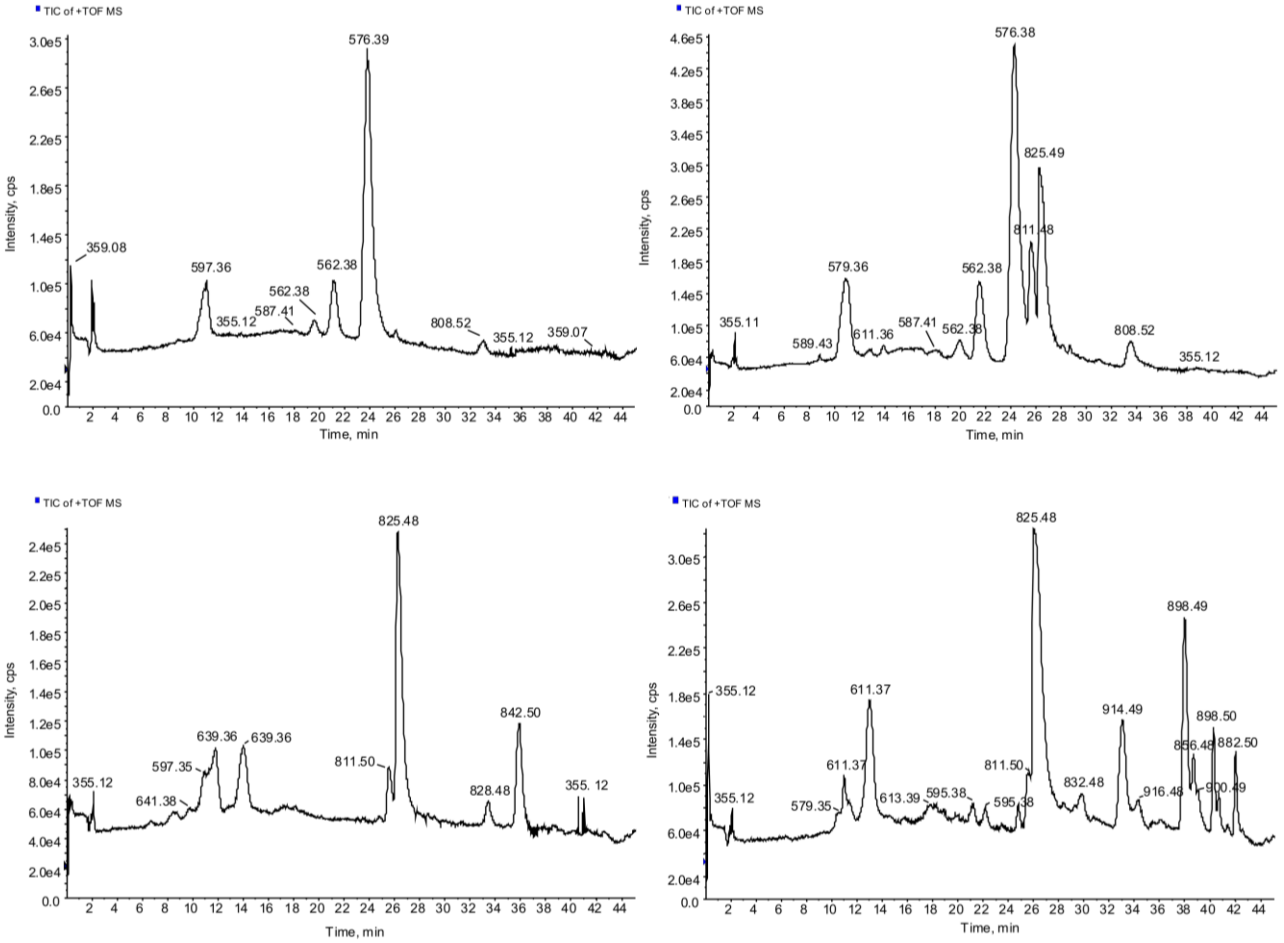

Figure S2. Enhanced ion product mass spectra of spumigin **12** Hpla-Tyr-Pro-Argal with [M + H] ion at *m/z* 583 (*N. spumigena* 1403). The structure was elucidated mainly based on the mass signals at *m/z*: 565 [M + H − H_2O], 523 [M + H − H_2O − CH_2N_2], 425 [Hpla-Tyr-Pro + H], 300 [Hpla-Tyr + H − CO], 239 [Pro-Argal + H − NH_2], 221 [Pro-Argal + H − H_2O − NH_2], 142 [Argal + H − NH_3], 136 Tyr-immonium ion, 100 [$C_4H_{10}N_3$] Argal fragment, 70 Pro-immonium ion.

Acq. Time: 10:19 Polarity/Scan Type: Positive Enhanced Product Ion Acq. File: 20101202.wiff
Acq. Date: Friday, December 03, 2010

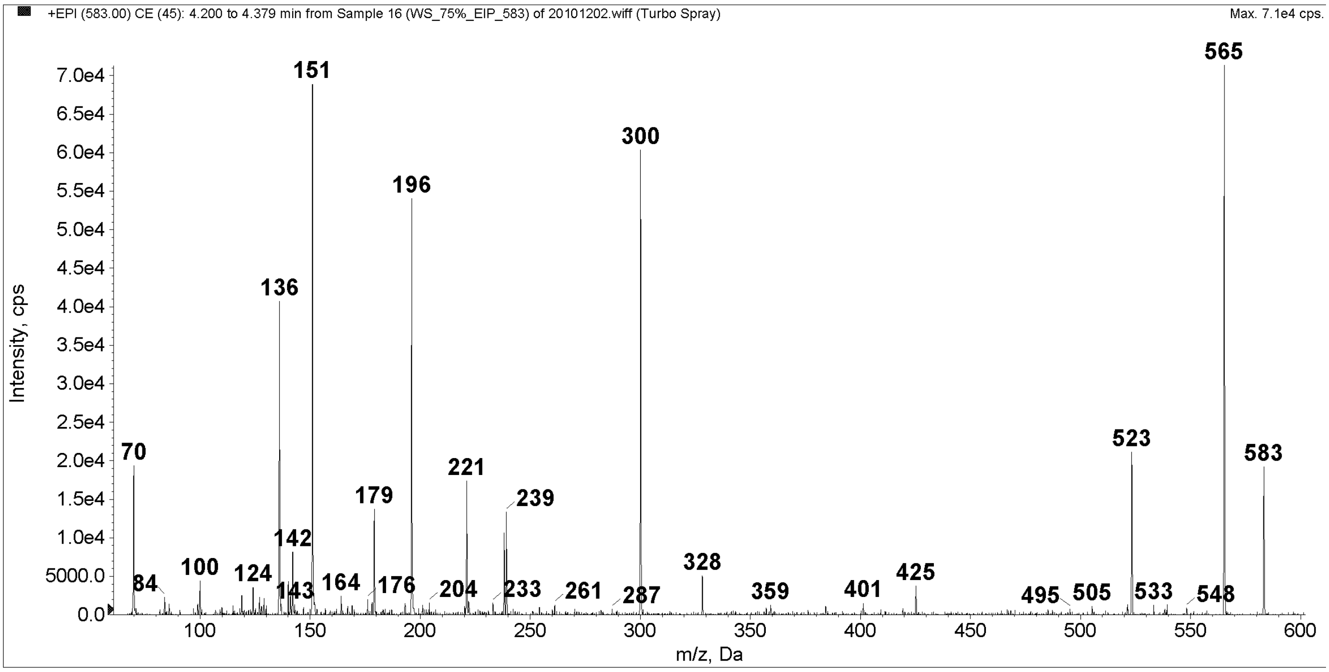

*Install Page 1 of 1 *FSE: AB Service

Figure S3. Enhanced ion product mass spectra of spumigin **13** Hpla-Hty-MePro-Agm with [M + H] ion at *m/z* 583 (*N. spumigena* Nodh2). The structure was elucidated mainly based on the mass signals at *m/z*: 565 [M + H − H_2O], 541 [M + H − CH_2N_2], 523 [M + H − H_2O − CH_2N_2], 453 [Hpla-Hty-MePro + H], 342 [Hpla-Hty + H], 314 [Hpla-Hyr + H − CO], 225 [MePro-Agm + H − NH_2], 208 [HOCHCO-Hty + H − CO], 114 [$C_5H_{12}N_3$] (Agm), 107 [CH_2PhOH], 84 MePro-immonium ion.

Acq. Time: 13:29 Polarity/Scan Type: Positive Enhanced Product Ion Acq. File: 20110729.wiff
Acq. Date: Friday, July 29, 2011

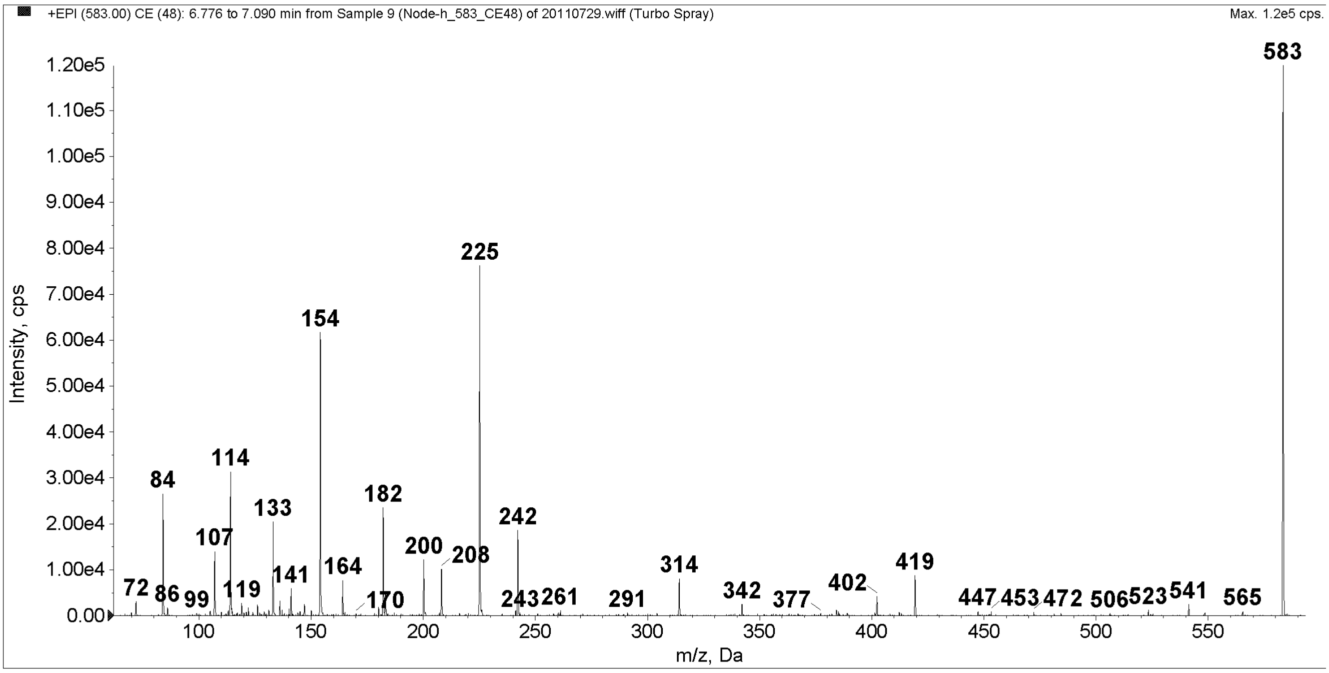

*Install Page 1 of 1 *FSE: AB Service

Figure S4. Enhanced ion product mass spectra of spumigin **15** (Hpla + 42)-Leu-Pro-Argal with [M + H] ion at *m/z* 575 (*N. spumigena* B15a). The structure was elucidated mainly based on the mass signals at *m/z*: 557 [M + H − H_2O], 515 [M + H − H_2O − CH_2N_2] or/and [M + H − CH_3COOH], 417 [(Hpla + 42)-Leu-Pro + H], 320 [(Hpla + 42)-Leu + H], 292 [(Hpla + 42)-Leu + H − CO], 239 [Pro-Argal + H − NH_2], 221 [Pro-Argal + H − H_2O − NH_2] 142 [Argal + H − NH_3], 100 [$C_4H_{10}N_3$] Argal fragment, 86 Leu-immonium ion, 70 Pro-immonium ion.

Acq. Time: 15:31 Polarity/Scan Type: Positive Enhanced Product Ion Acq. File: 20100309.wiff
Acq. Date: Tuesday, March 09, 2010

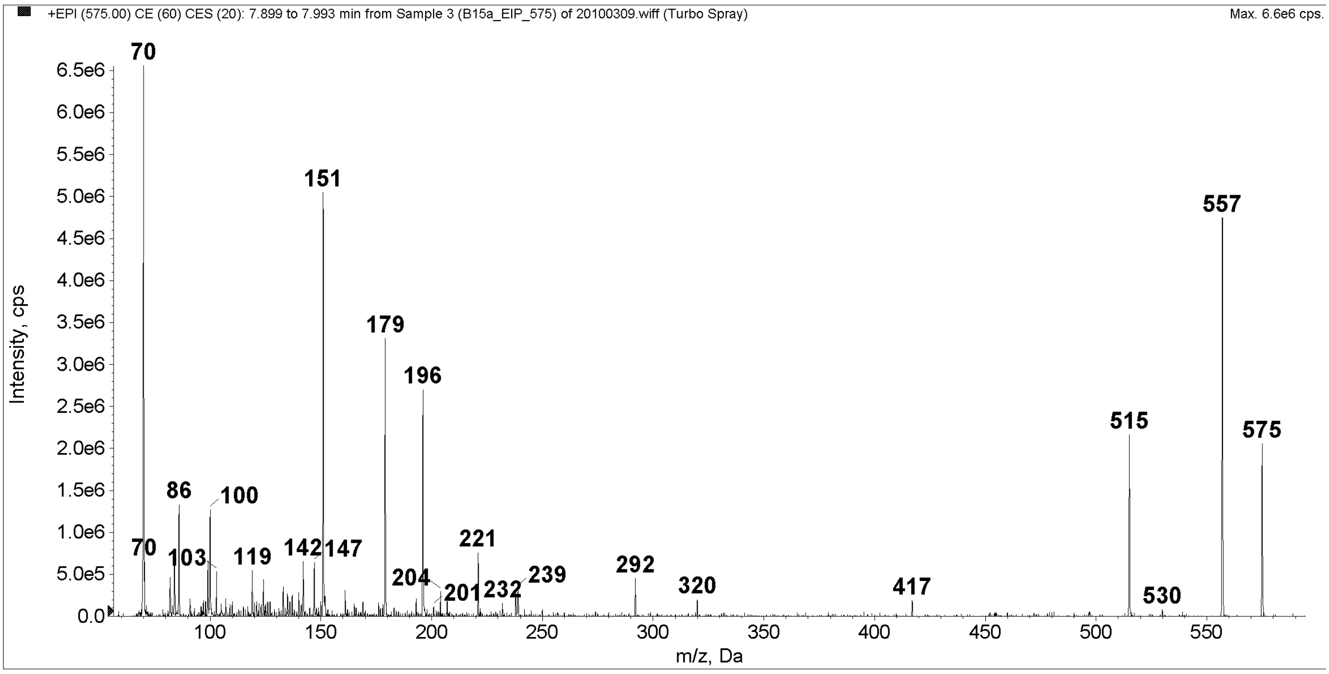

*Install Page 1 of 1 *FSE: AB Service

Figure S5. Enhanced ion product mass spectra of spumigin **18** Hpla-Hty-Pro-OH with [M + H] ion at *m/z* 457 (*N. spumigena* CCNP1403). The structure was elucidated mainly based on the mass signals at *m/z*: 439 [Hpla-Hty-Pro + H], 342 [Hpla-Hty + H], 314 [Hpla-Hyr + H − CO], 208 [HOCHCO-Hty + H − CO], 107 [CH_2PhOH], 70 Pro-immonium ion.

Acq. Time: 13:06 Polarity/Scan Type: Positive Enhanced Product Ion Acq. File: 20101202.wiff
Acq. Date: Thursday, December 02, 2010

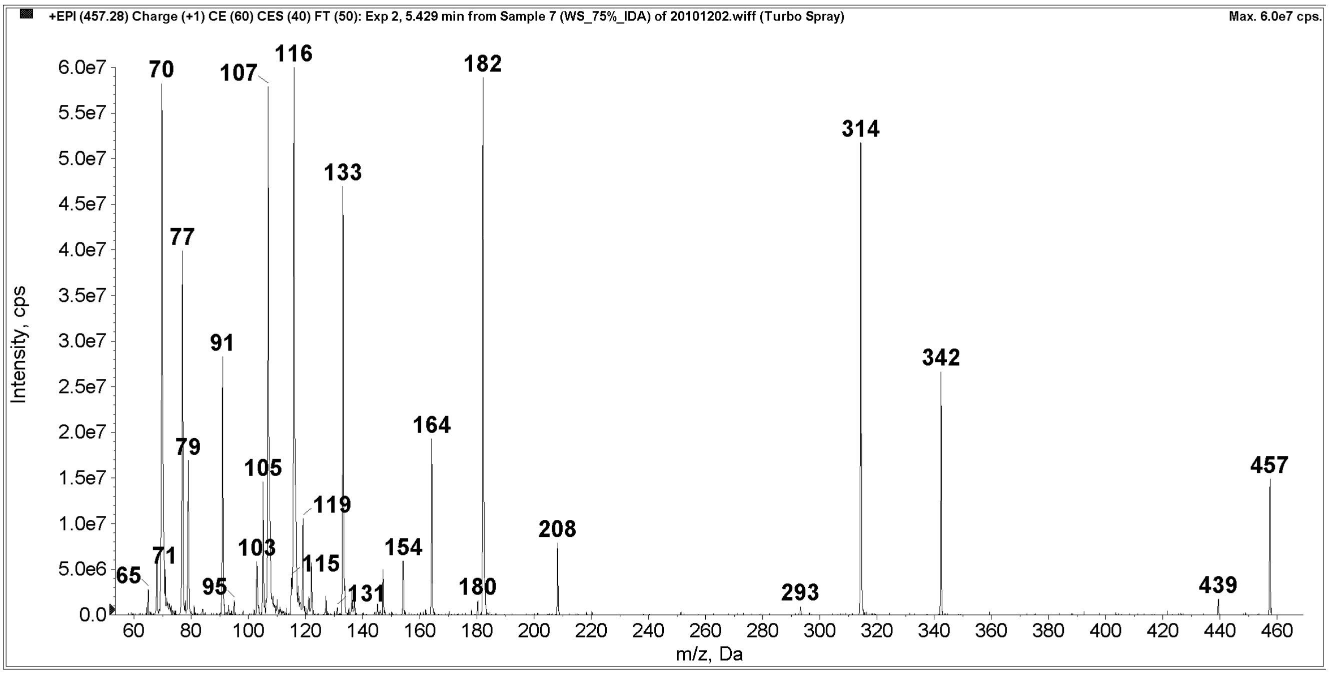

*Install Page 1 of 1 *FSE: AB Service

Figure S6. Enhanced ion product mass spectra of partially identified aeruginosin **19** with [M + H] ion at *m/z* 603 (*N. spumigena* KAC66). The mass signals were assigned to the following fragments: 585 [M + H − H_2O], 429 [M + H − Arg], 342 [Choi-Arg + H], 325 [Choi-Arg + H − NH_2], 307 [Choi-Arg + H − H_2O − NH_3], 300 [Choi-Arg + H − CH_2N_2]), 282 [Choi-Arg + H − H_2O − CH_2N_2], 175 [Arg + H], 140 Choi-immonium ion, 122 Choi-immonium − H_2O.

Acq. Time: 09:17 Polarity/Scan Type: Positive Enhanced Product Ion Acq. File: 20100319SET1.wiff
Acq. Date: Friday, March 19, 2010

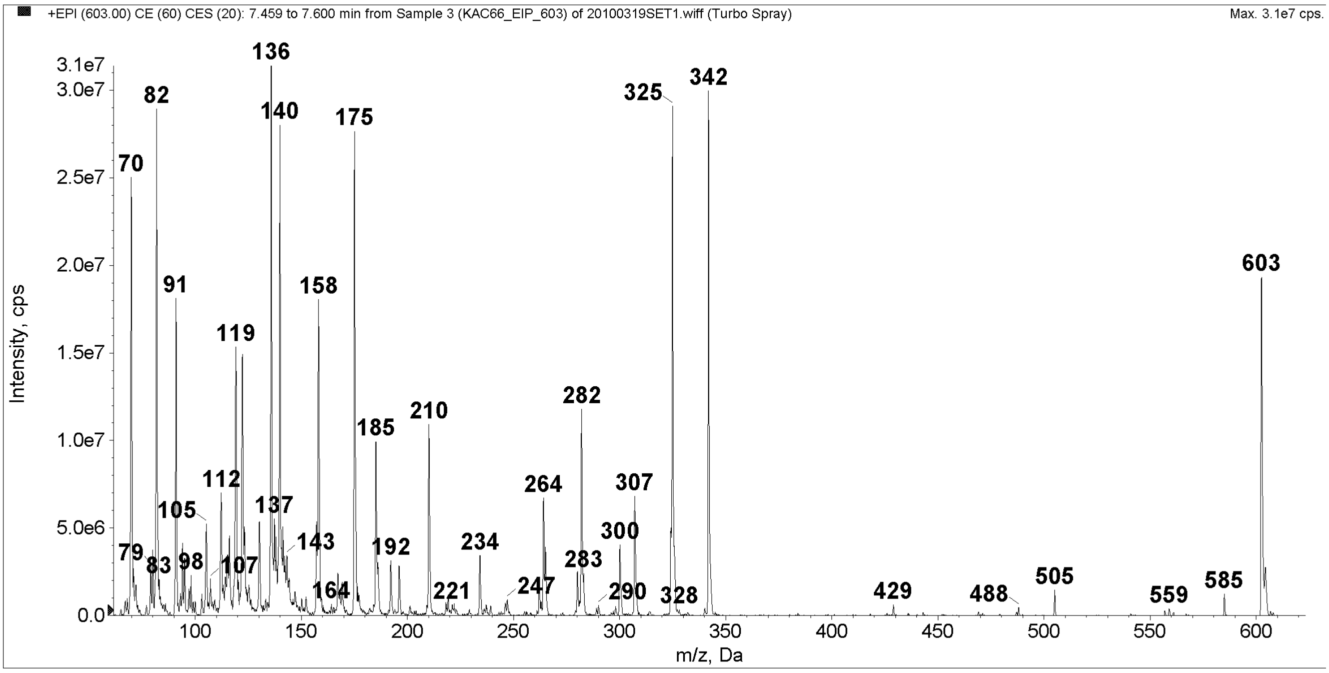

*Install Page 1 of 1 *FSE: AB Service

Figure S7. Enhanced ion product mass spectra of partially identified aeruginosin **22** with [M + H] ion at *m/z* 559 (*N. spumigena* Node2). The mass signals were assigned to the following fragments: 541 [M + H − H_2O], 298 [Choi-Agm + H], 281 [Choi-Agm + H − NH_2], 256 [Choi-Arg + H − CH_2N_2], 238 [Choi-Arg + H − H_2O − CH_2N_2], 210 [Choi-Arg + H − H_2O − CH_2N_2 − CO], 140 Choi-immonium ion, 122 Choi-immonium − H_2O, 114 [$C_5H_{12}N_3$] (Agm).

Acq. Time: 11:10 Polarity/Scan Type: Positive Enhanced Product Ion Acq. File: 20110822.wiff
Acq. Date: Monday, August 22, 2011

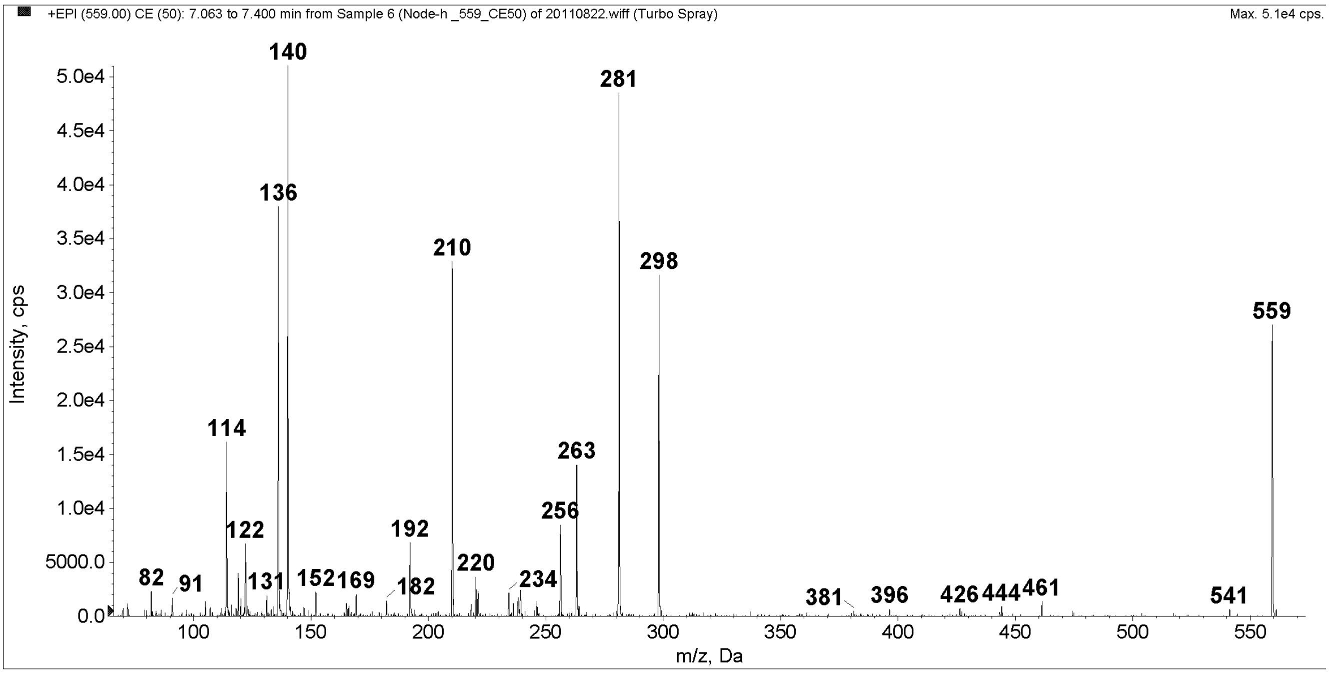

*Install Page 1 of 1 *FSE: AB Service

Figure S8. Enhanced ion product mass spectra of anabaenopeptin **25** Phe-CO-[Lys-Val-Hty-MeHty-MetO] with [M + H] ion at *m/z* 934 (*N. spumigena* bloom sample, 3 July 2012). The mass signals were assigned to the following fragments: 916 [M + H − H_2O], 906 [M + H − CO], 888 [M + H − H_2O − CO], 870 [M + H − CH_3SOH (from MetO)], 787 [M + H − MetO], 769 [M + H − Phe - 2H], 757 [M + H − Hty], 705 [M + H − Phe − 2H − CH_3SOH (from MetO)], 677 [M + H − (CO-Phe) − 2H − CH_3SOH (from MetO)], 594 [Val-(Lys-CO)-MetO-MeHty + H], 511 [MeHty-MeO-(Lys-CO) + H], 447 [MeHty-MeO-(Lys-CO) + H − CH_3SOH (from MetO)], 403 [Lys-Val-Hty-H], 369 [MeHty-Hty + H], 339 [MetO-MeHty + H], 164 MeHty, 107 [CH_2PhOH], 84 Lys-immonium ion.

Acq. Time: 09:43 Polarity/Scan Type: Positive Enhanced Product Ion Acq. File: 20120711.wiff
Acq. Date: Wednesday, July 11, 2012

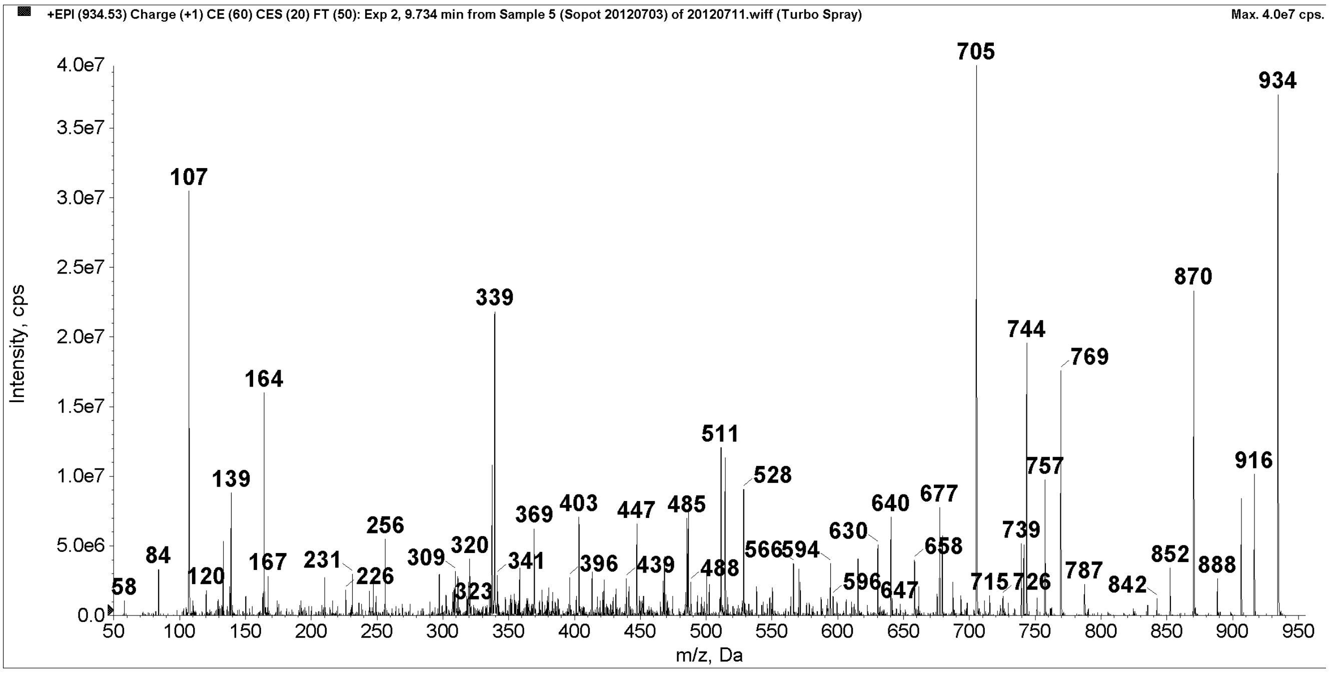

*Install Page 1 of 1 *FSE: AB Service

Figure S9. Enhanced ion product mass spectra of anabaenopeptin **32** Ile-CO-[Lys-MetO-Hph-MeHty-MetO] with [M + H] ion at *m/z* 932 (*N. spumigena* CCNP1402). The mass signals were assigned to the following fragments: 914 [M + H − H_2O], 904 [M + H − CO], 886 [M + H − H_2O − CO], 868 [M + H − CH_3SOH (from MetO)], 801 [M + H − Ile − 2H], 737 [M + H − Ile − 2H − CH_3SOH (from MetO)], 511 [MeHty-MeO-(Lys-CO) + H], 447 [MeHty-MeO-(Lys-CO) + H − CH_3SOH (from MetO)], 436 [MeHy-Hph-MetO + H − CH_3SOH (from MetO)], 339 [MeHty-MetO + H], 164 MeHty, 107 [CH_2PhOH], 84 Lys-immonium ion.

Acq. Time: 11:15 Polarity/Scan Type: Positive Enhanced Product Ion Acq. File: 20120126.wiff
Acq. Date: Thursday, January 26, 2012

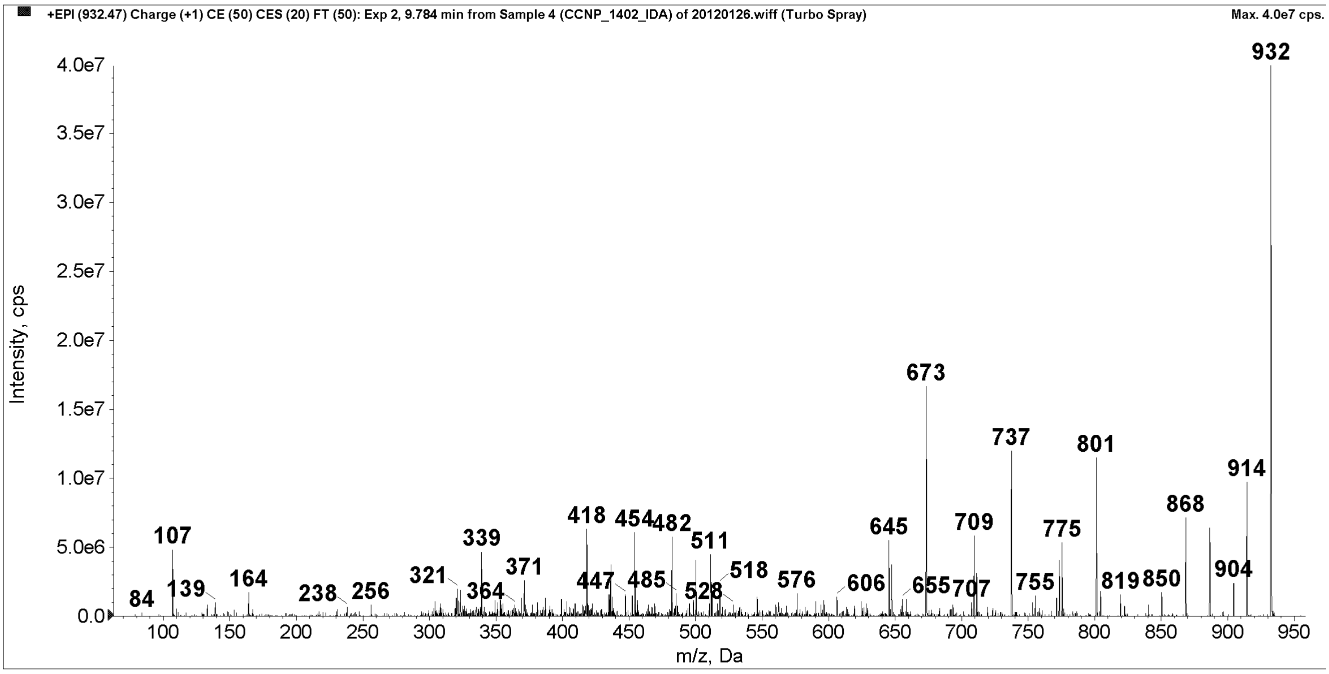

*Install Page 1 of 1 *FSE: AB Service

Figure S10. Enhanced ion product mass spectra of anabaenopeptin **39** Ile-CO-[Lys-Met-Hph-MeHph-AcSer] with [M + H] ion at *m/z* 882 (*N. spumigena* BY1). The mass signals were assigned to the following fragments: 864 [M + H − H_2O], 854 [M + H − CO], 822 [M + H − CH_3COOH (from AcSer)], 751 [M + H − Ile − 2H], 725 [M + H − (CO-Ile)], 723 [M + H − (CO-Ile) − 2H], 530 [Met-(Lys-CO-Ile)-Met + H − H_2O], 486 [Met-(Lys-CO-Ile)-(AcSer) + H − CH_3COOH (from AcSer)], 477 [MeHph-AcSer-(Lys-CO) + H], 468 [MeHph-Hph-Met + H], 337 [Hph-MeHph + H], 84 Lys-immonium ion.

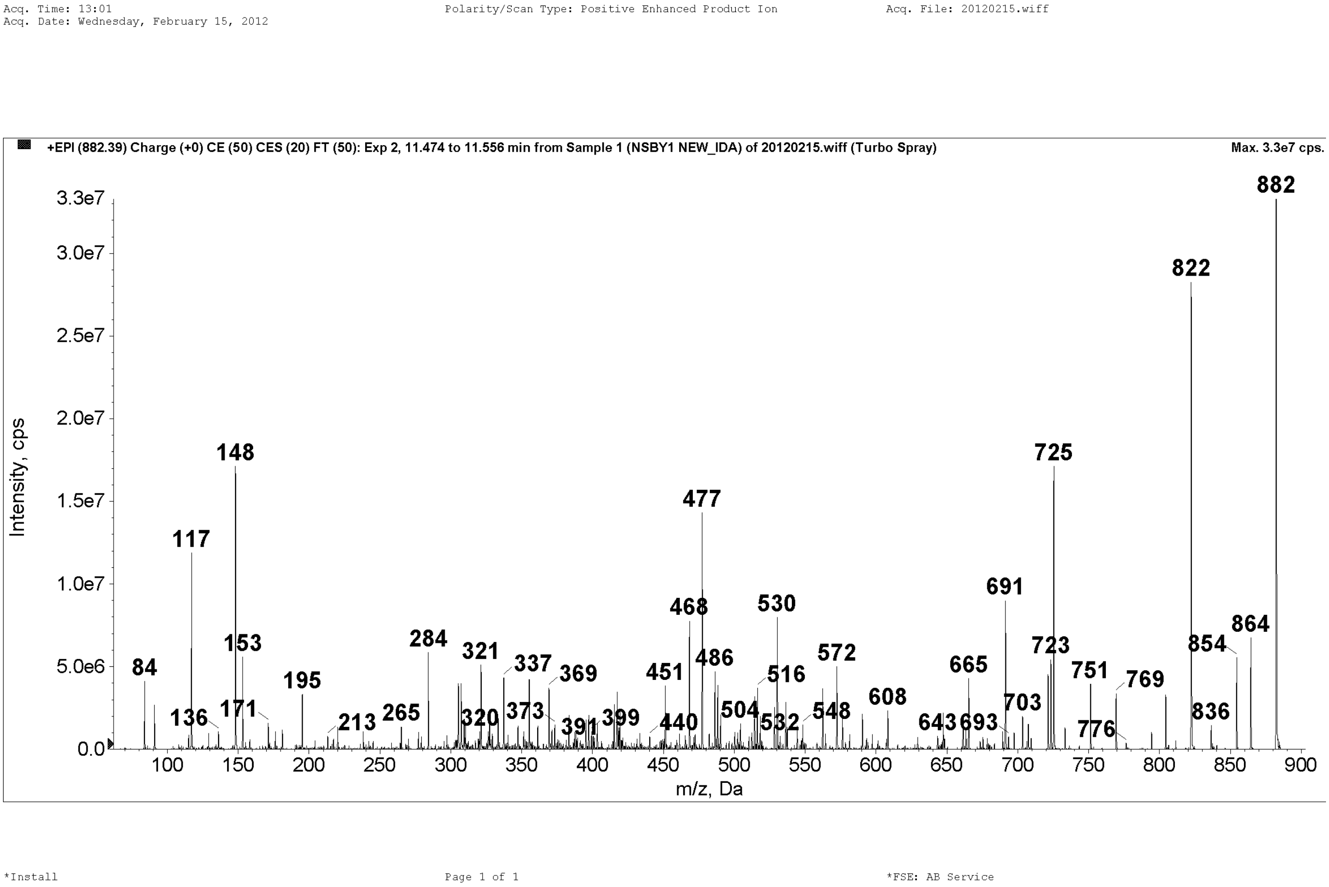

Figure S11. Enhanced ion product mass spectra of anabaenopeptin **41** Ile-CO-[Lys-Ile-Hph-MeHty-AcSer] with [M + H] ion at *m/z* 880 (*N. spumigena* Node2). The mass signals were assigned to the following fragments: 862 [M + H − H_2O], 852 [M + H − CO], 834 [M + H − H_2O − CO], 820 [M + H − CH_3COOH (from AcSer)], 767 [M + H − Ile], 749 [M + H − Ile − 2H], 723 [M + H − (CO + Ile)], 719 [M + H − Hph], 606 [MeHty-AcSer-(Lys-CO-Ile) + H], 493 [MeHty-AcSer-(Lys-CO) + H], 164 MeHty, 107 [CH_2PhOH], 84 Lys-immonium ion.

Acq. Time: 12:53 Polarity/Scan Type: Positive Enhanced Product Ion Acq. File: 20120126.wiff
Acq. Date: Thursday, January 26, 2012

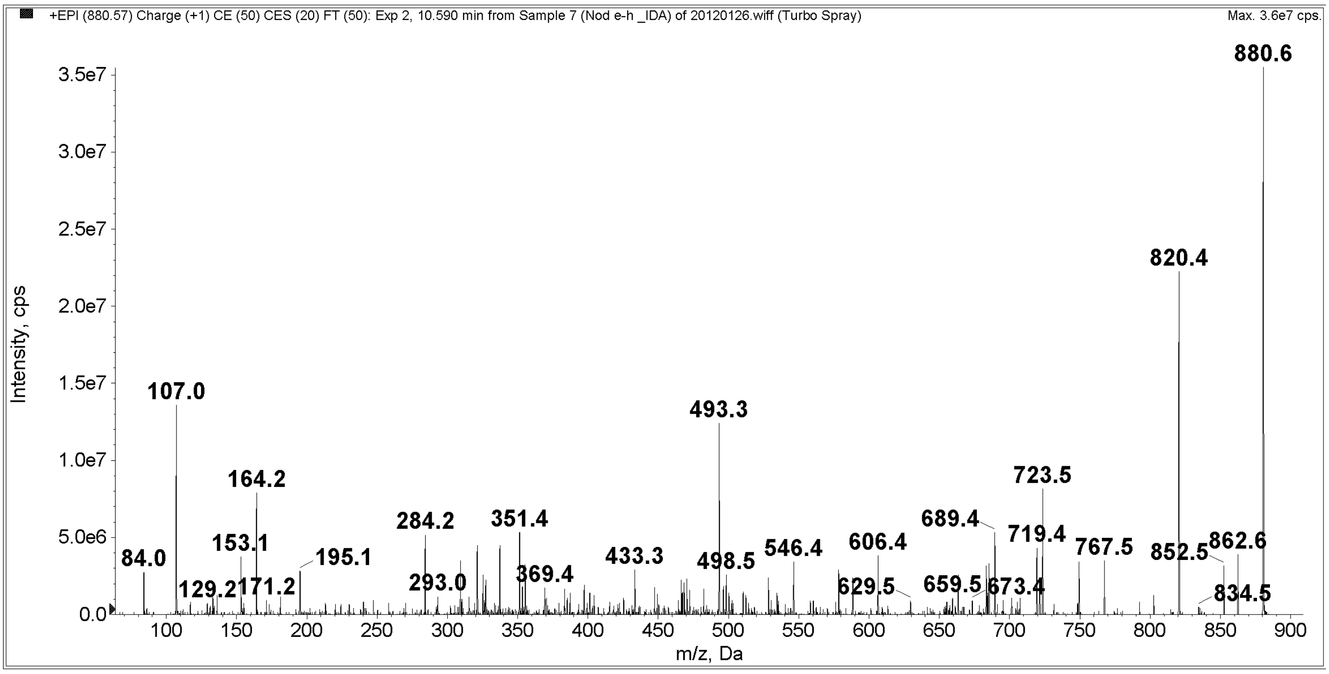

*Install Page 1 of 1 *FSE: AB Service
